# Pharmacist intervention to improve adherence to medication among heart failure patients at North East Ethiopia hospital

**DOI:** 10.1002/prp2.1199

**Published:** 2024-04-30

**Authors:** Abate Wondesen Tsige, Tsegaye Ababiya Kotiso, Kassahun Dires Ayenew, Siraye Genzeb Ayele

**Affiliations:** ^1^ Department of Pharmacy, College of Health Sciences Debre Berhan University Debre Berhan Ethiopia; ^2^ Department of Pharmacy Tirunesh‐Beijing Hospital Addis Ababa Ethiopia; ^3^ Department of Midwifery, School of Nursing and Midwifery, College of Health Sciences Addis Ababa University Addis Ababa Ethiopia

**Keywords:** adherence, Debre Berhan comprehensive specialized hospital, Ethiopia, heart failure

## Abstract

Heart failure (HF) is a major and growing medical problem and its management is still challenging due to the coexistence of complications, co‐morbidity, and medication non‐adherence. HF patients who are adherent to their medication have fewer HF exacerbations, improved survival, and lower healthcare expenditure. Adherence to HF medication plays a pivotal role in attaining maximal therapeutic outcomes. The aim was to assess the medication adherence of heart failure patients at Debre Berhan Comprehensive Specialized Hospital (DBCSH). A pre‐post interventional study was undertaken from July 1, 2022, to December 31, 2022, at the medical referral clinic of DBCSH. The educational interventions were provided for 6 months. Medication adherence was determined using the Morisky Green Levin Medication Adherence Scale (MGLS). The data was entered into Epidata version 4.2.0 and analyzed using SPSS version 25.0 statistical software. Descriptive statistics and binary logistic regression analysis were performed. The strength of the association between predictor variables and outcome variables was determined using a 95% confidence interval and adjusted odd ratio. In the pre‐intervention phase, 54.6% of patients had medium medication adherence, while in the post‐intervention phase, 36.4% of patients had high medication adherence and 61.9% of patients had medium medication adherence. Following the intervention, medication cost (120, 50%), inadequate availability of drugs (75, 31%), and forgetfulness (30, 13%) were the main reasons for medication non‐adherence. The presence of co‐morbidity and the number of co‐morbidity (*p* < .05) were significantly associated with the occurrence of decreased medication adherence in the pre‐intervention phase. Interventions by pharmacists to educate HF patients about the nature of their disease and providing brochures to increase awareness of their medications have been shown to improve medication adherence.

**Abbreviations**
ADHF: Acute decompensated heart failure
CVDs: Cardiovascular diseases
DBCSH: Debre Berhan Comprehensive Specialized Hospital
DDI: Drug‐drug interaction
EF: Ejection fraction
HF: Heart failure
HMIS: Health Management Information Systems
MGLS: Morisky Green Levin Medication Adherence Scale
OTC: Over‐the‐counter
SPSS: Statistical Package for Social Sciences
WHO: World Health Organization

## 1 INTRODUCTION

Globally, cardiovascular diseases (CVDs) are the leading cause of death and an estimated 17.9 million people died from CVDs in 2019, over three‐quarters of which took place in low‐ and middle‐income countries. In 2019, out of the 17 million premature deaths (under the age of 70) due to non‐communicable diseases, 38% were caused by CVDs.^1^

In Ethiopia, there is little evidence about the burden of CVD. A study conducted using the 2017 global burden of disease, injuries, and risk factors (GDB) reported that CVD prevalence was 5534 per 100 000 population.^2^

Heart failure (HF) is one of the most prevalent types of CVD, resulting in a major medical and economic problem.^3^ It is a progressive clinical syndrome that can result from any changes in cardiac structure or function that impair the ability of the ventricle to fill with or eject blood.^4^ The incidence and prevalence of HF are increasing, approximately 6.5 million Americans have HF with 1 000 000 new cases diagnosed each year, with over 30 billion US dollars in annual expenditures.^5^ Based on the phenotypes of the disease, HF can be classified as HF with a preserved ejection fraction (EF) (HFpEF), HF with a reduced EF (HFrEF), acute decompensated HF (ADHF), and advanced HF.^6^

Many patient factors should be considered when selecting HF medications. These factors include compelling indications, co‐morbid disease states, concomitant medications use, cost of medication, and medication adherence.^7^

Heart failure management is still challenging due to the coexistence of complications and co‐morbidity.^8^ In addition, prescribing a higher number of medications for HF patients has also been associated with negative health outcomes, frequent hospitalization, waste of resources, and patient non‐adherence to their drug regimens.^9^ So, HF guidelines recommend adherence to prescribed medications and optimized self‐care measures are useful for HF patient prognosis.^10^, ^11^

Adherence is defined by the World Health Organization (WHO) as “the extent to which a person's behavior taking medication, following a diet, and/or executing lifestyle changes, corresponds with agreed recommendations from a health care provider”.^12^, ^13^ Heart failure patients who were adherent to their medications improved patient clinical outcomes.^14^

Various studies conducted in different countries indicated that HF patients who are adherent to their medication had fewer HF exacerbations, improved survival, fewer emergency department visits, and lower healthcare expenditure.^15^, ^16^, ^17^ Non‐adherence to HF medication is associated with increased hospitalization,^18^, ^19^ worsening symptoms, increased disease progression, and an overall increase in healthcare costs^18^ and emergency visits.^20^ Studies conducted elsewhere using different assessment tools of medication adherence showed HF medication adherence rates are consistently suboptimal across studies.^12^, ^14^, ^21^, ^22^, ^23^, ^24^, ^25^, ^26^

To reach targeted therapeutic outcomes and reduce medication non‐adherence issues of HF patients, pharmacist‐led implementation of medication therapy management (MTM) service has paramount importance.

Medication therapy management services are pharmacist‐provided non‐dispensing services that aim to address the drug‐related needs of the patient, improve medication adherence, patient counseling, motivational interviewing, patient assessment, patient education, documentation, follow‐up, and assist the patient in achieving therapeutic goals in collaboration with other healthcare professionals.^27^, ^28^, ^29^

A prospective interventional randomized control trial conducted in the USA showed, 67.9% (*n* = 122) and 78.8% (*n* = 192) of medication adherence in the usual care and the intervention groups, respectively.^30^ In addition, another prospective interventional study reported, 58.5% (*n* = 139) and 75.7% (*n* = 140) of medication adherence in the control and the intervention group, respectively.^31^

Studies are available on the implementation of pharmacist intervention in different developed countries. However, there are no studies conducted in resource‐constrained countries such as Ethiopia. This study aimed to assess pharmacist interventions to improve medication adherence of HF patients at the medical referral clinic of DBCSH, Ethiopia.

## 2 MATERIALS AND METHODS

### 2.1 Study setting, design, and population

A pre‐post interventional study was undertaken from July 1, 2022, to December 30, 2022, at DBCSH. The medical referral clinic is one of the specialty clinics in DBCSH providing cardiac care. All ambulatory HF patients who attended the medical referral clinic of DBCSH ≥18 years old and HF patients who had complete medical records were included in the study. Patients who could not stand the interview and patients who changed follow‐up sites were excluded from the study.

### 2.2 Sample size determination and sampling technique

The sample size was estimated using a single population proportion formula.^32^ Using a 50% prevalence of medication adherence, and adding 10% of the contingency, the calculated final sample size was 423.

Study participants were selected using a systematic random sampling technique using the Health Management Information Systems (HMIS) list of HF patients at a medical referral clinic of DBCSH on each day of the data collection period.

### 2.3 Data collection procedures and tools

Study participant's demographic and clinical data were collected using a pre‐tested structured questionnaire and data abstraction tools, respectively. Trained two clinical pharmacists and three nurses carried out the data collection. The clinical pharmacists were involved in clinical data review and patient education, and the nurses were involved in patient interviews.

Medication adherence was determined using the Morisky Green Levin Medication Adherence Scale (MGL). It has four items focusing on past medication use patterns with closed dichotomies (yes/no). Each “no” response was rated as 1 and each “yes” response was rated as 0. The total summed score ranges from 0 to 4 and was grouped into high adherence (0 points scored), medium adherence (1–2 points scored), and poor adherence (≥3 points scored).^33^

### 2.4 Interventions

On the day of the patient appointment, patient demographic data and pre‐intervention medication adherence data were collected. We provided education services about over‐the‐counter (OTC) drug use, diet modifications (including salt restriction), physical activity, and self‐monitoring of weight. In addition, based on the standard HF treatment guidelines^10^, ^34^, ^35^ trained data collectors provide verbal education about the importance of medication adherence.

Study participants received brochures that contained specific information about HF and HF medications as an intervention package to improve medication adherence and lifestyle modifications.

Time spent with each participant was 16–21 min for a medication review and interview, and 14–21 min for intervention and documentation both in pre‐ and post‐intervention phases.

### 2.5 Outcome measures

The change in medication adherence rate was the main outcome measured from pre‐intervention (baseline) to post‐intervention. The change in medication adherence rate was determined by MGLS.^33^

### 2.6 Data analysis and interpretations

The collected data were sorted, cleaned, coded, and entered into Epidata version 4.2.0 and then exported to Statistical Package for Social Sciences (SPSS) version 25 software. Descriptive statistics were used to summarize frequencies, means, and percentages. Binary logistic regression analysis was performed to show the association between predictors' variables with medication adherence. All variables with *p* < .25 in the univariable binary logistic regression analysis were included in the multivariable binary logistic regression analysis, which was performed to determine the potential predictors of medication adherence. A *p*‐value of <.05 was considered statistically significant.

## 3 RESULTS

### 3.1 Socio‐demographic characteristics of study participants

As per the eligibility criteria, 11 study participants were excluded out of 423 recruited patients. The mean age of study participants was 44.57 (SD, 17.4) years and most of them were in the age range of 18–93 years. The majority of the study participants were paid out of pocket for their medication (54.6%). About 60.4% of participants resided in urban areas (Table 1).

**TABLE 1:** Socio‐demographic characteristics of heart failure patients from July to December 2022.

Variables	Categories	Number (%)	Mean ± SD	Range
Sex	Male	191 (46.4)		
	Female	221 (53.6)		
Age (years)	18–33	136 (33)		
	34–49	103 (25)	44.57 ± 17.14	18–93 years
	50–65	126 (30.6)		
	>65	47 (11.4)		
BMI (kg/m^2^)		*n* = 340		
	BMI < 18.5	29 (8.5)		
	BMI 18.5–24.9	203 (59.7)	22.9 ± 3.26	
	BMI 25–30	95 (27.9)		
	BMI ≥30	13 (3.9)		
Religion	Orthodox	274 (66.5)		
	Muslim	81 (19.7)		
	Catholic	17 (4.1)		
	Protestant	40 (9.7)		
Marital status	Single	121 (29.4)		
	Married	265 (64.3)		
	Widowed	5 (1.2)		
	Divorced	21 (5.3)		
Educational status	Only read and write	110 (26.7)		
	Primary	116 (28.2)		
	Secondary	96 (23.3)		
	Diploma and above	90 (21.8)		
Place of residence	Addis Ababa	249 (60.4)		
	Out of Addis Ababa	163 (39.6)		
Occupation	Governmental Employed	118 (28.6)		
	Private employed	105 (25.5)		
	Unemployed (housewife, student)	96 (23.3)		
	Self‐employed (farmer, daily labor, merchant, driver)	59 (14.3)		
	Others^a^	34 (8.3)		
Source of medications	Free	85 (20.6)		
	Paid	225 (54.6)		
	Covered by insurance	52 (12.6)		
	Covered by family	50 (12.1)		

^a^ Retired.

### 3.2 Clinical characteristics

The greater proportion of patients (33.5%, 138) had 5–10 years of HF duration. The mean duration of HF treatment was 9.45 years (SD, 6.47) with a range of 1–35 years. More than one third of patients (30.6%, 126) did not have co‐existing co‐morbidities and nearly one fourth of patients (23.5%, 97) had three co‐morbidities. More than half of the patients (56.8%, 234) had taken 2–4 drugs (Table 2).

**TABLE 2:** Clinical characteristics of heart failure patients from July to December 2022.

Clinical characteristics	Category	Number (%)	Mean ± SD	Range
Duration of HF (years)	<5 years	134 (32.5)	9.45 ± 6.47	1–35 years
	5–10 years	138 (33.5)		
	>10 years	140 (34)		
Admission history	Yes	182 (44.2)		
	No	230 (55.8)		
Number of drugs	2–4 drugs	234 (56.8)	4.2 ± 1.71	2–8 drugs
	≥5 drugs	178 (43.2)		
Frequency of encountered for MTM visit	≥ 2 months	245 (59.47)		
	≥3 months	167 (40.53)		
Drug allergy history	Yes	16 (3.9)		
	No	396 (96.1)		
Drug–drug interaction	Yes	154 (37)		
	No	258 (63)		
Co‐morbidity	Yes	126 (30.6%)		
	No	286 (69.4%)		
Number of co‐morbidity	One	83 (20.1%)		
	Two	48 (11.7%)		
	Three	58 (14.1%)		
	Four and above	97 (23.5%)		

Nearly two thirds, (63.6%, 262) of the study participants had no agreed exercise plan with physicians. The majority of patients (73.1%, 301) had agreed on dietary plans with physicians.

Study participants had received an average of four drugs. Diuretics (26%) were the most commonly prescribed medications followed by B‐blocker (16%).

### 3.3 Medication adherence

In the pre‐intervention, 54.6% of study participants had medium medication adherence and 39.1% had low medication adherence. However, in the post‐intervention, 38.4% of patients had high medication adherence (*p* < .001) and 61.9% of patients had medium medication adherence (*p* < .002) (Table 3).

**TABLE 3:** Rates of heart failure patients' medication adherence from July to December 2022.

Rates of medication adherence	Participants		*p*‐value^⁎^
	Pre‐test (*N* %)	Post‐test (*N* %)	
Poor adherence	161 (39.1%)	7 (1.7%)	<.001
Medium adherence	225 (54.6%)	255 (61.9%)	.002
High adherence	26 (6.3%)	150 (36.4%)	<.001

^⁎^ Paired samples *t*‐test.

### 3.4 Reasons for medication non‐adherence

Forgetfulness (40%) was the main reason, followed by inadequate availability of medications (29%), and the cost of medication too expensive (22%) in the pre‐intervention. Following the intervention, forgetfulness came down to 13%, although medication cost (50%, 120) went up and inadequate availability (31%, 75) remained almost similar (Table 4).

**TABLE 4:** Reasons for medication non‐adherence of heart failure patients from July to December 2022.

Reason for medication non‐adherence	Participants	
	Pre‐test (*N* %)	Post‐test (*N* %)
The cost of medication is too expensive	70 (22)	120 (50)
Inadequate availability of medication	90 (29)	75 (31)
Drug side effect	3 (1)	4 (2)
Simply forgetfulness	125 (40)	30 (13)
Patients have no support at home	1 (0.1)	1 (0.1)
Regimen complexity	20 (7)	8 (3)
The patient prefers not to take	1 (0.1)	0
Patients felt worse when taking a drug	4 (1)	1 (0.5)

### 3.5 Predictors of medication adherence

From clinical and socio‐demographic characteristics incorporated in the logistic regression analysis, the number of co‐morbidities, number of drugs prescribed, and the presence of drug–drug interaction(DDI) were significantly associated with medication adherence (*p* < .05) in the post‐intervention, whereas, the presence of co‐morbidity and number of co‐morbidity were significantly associated with medication adherence in the pre‐intervention (Table 5).

**TABLE 5:** Univariable and multivariable analysis of factors associated with medication adherence of heart failure patients from July to December 2022.

Variables	Categories	Pre‐test		Post‐test	
		Odds Ratios (95% CI)		Odds Ratios (95% CI)	
		COR	AOR	COR	AOR
Sex	Male	1	1	1	1
	Female	0.98 (0.66–1.5)	0.99 (0.65–1.54)	0.85 (0.55–1.33)	1.12 (0.65–1.91)
Age(years)	18–33	1	1	1	1
	34–49	0.71 (0.42–1.2)	1.24 (0.63–2.47)	1.11 (0.59–2.07)	1.25 (0.51–3.07)
	50–65	0.67 (0.41–1.1)	1.35 (0.68–2.68)	0.63 (0.36–1.11)	1.99 (0.79–5.02)
	>65	0.7 (0.36–1.4)	1.46 (0.62–3.47)	0.55 (0.27–1.13)	1.39 (0.60–3.26)
Adverse drug reaction	No	1	1	1	1
	Yes	2.88 (0.79–1.63)	2.53 (0.68–9.39)	1.16 (0.36–3.78)	1.31 (0.33–5.26)
Drug–drug interaction	No	1	1	1	1
	Yes	1.1 (0.75–10.47)	1.24 (0.81–1.89)	0.43 (0.27–0.68)	**0.62 (1.02–3.13)**
Occupation	Governmental Employed	1	1	1	1
	Private employed	0.75 (0.44–1.3)	0.71 (0.40–1.25)	0.70 (0.38–1.30)	1.87 (0.69–5.12)
	Unemployed	0.68 (0.39–1.2)	0.60 (0.33–1.11)	0.69 (0.37–1.29)	1.22 (0.45–3.31)
	Self‐employed	0.82 (0.44–1.53)	0.52 (0.26–1.10)	0.95 (0.45–2.03)	1.25 (0.45–3.50)
	Others^a^	0.56 (0.26–1.24)	0.65 (0.28–1.51)	0.49 (0.22–1.13)	1.44 (0.46–4.49)
Source of medications	Free	1	1	1	1
	Paid	0.88 (0.54–1.51)	0.88 (0.51–1.52)	0.96 (0.53–1.74)	0.72 (0.35–1.48)
	Covered by insurance	0.70 (0.35–1.42)	0.77 (0.36–1.65)	0.50 (0.23–1.07)	0.41 (0.16–1.01)
	Covered by family	0.69 (0.34–1.4)	0.61 (0.28–1.31)	0.61 (0.28–1.34)	0.48 (0.19–1.22)
Presence of co‐morbidity	No	1	1	1	1
	Yes	0.66 (0.43–0.99)	**0.69 (0.44–0.90)**	0.50 (0.29–0.85)	1.58 (0.84–2.95)
Number of co‐morbidity		0.89 (0.79–0.99)	**0.88 (0.76–0.99)**	0.55 (0.47–0.64)	**0.58 (0.48–0.69)**
Number of drugs	One	1	1	1	1
	2–4 drugs	0.63 (0.27–1.5)	0.64 (0.26–1.6)	0.95 (0.27–3.39)	0.97 (0.84–12.9)
	≥5 drugs	0.6 (0.3–1.4)	0.79 (0.31–2.04)	0.19 (0.05–0.65)	**0.32 (1.59–5.11)**

During pre‐intervention, patients who had co‐morbidity were 0.69 times less likely to be adherent to medication compared with patients who had no co‐morbidity (AOR = 0.68, 95% CI: 0.44–0.90). In addition, the number of co‐morbidity increased by one unit the patient medication adherence decreased by 0.88 times.

Following intervention patients who had DDI were 0.62 times less likely to be adherent than patients who had no drug–drug interaction (AOR = 0.62, 95% CI: 1.02–3.13). Patients who took ≥5 drugs were 0.32 times less likely to be adherent compared with patients who had taken one drug (AOR = 0.32, 95% CI: 1.59–5.22) (Table 5).

## 4 DISCUSSION

Adherence to HF medications remains the cornerstone of preventing avoidable readmissions, premature deaths, and unnecessary healthcare expenses.^14^

In the current study, a large number of HF patients adherent at the post‐intervention reflected in MGLS. Medication adherence of study participants was increased following the pharmacist's provision of intervention. This finding was similar to previous studies conducted elsewhere.^30^, ^36^, ^37^, ^38^, ^39^ In addition, this finding was supported by a systematic review and meta‐analysis done by Ruppar et al indicated pharmacist intervention had significant effects on improving HF patients' medication adherence.^40^ However, it was in contrast with a study conducted in Australia indicating pharmacists discussing with patients about their medicines did not improve medication adherence. This difference may be differences in setting, participant's clinical characteristics, methods used to measure medication adherence, study design, culture of pharmacists' approach to patients, religious, and educational background of participants. In addition, differences in socioeconomic status of study participants also may be contributing to medication non‐adherence.

In this study, we introduced a pharmacist‐led intervention to improve medication adherence. The main focus of the intervention was to resolve the problems identified in the pre‐intervention phase. The patients were educated to improve awareness of their treatment, the importance of medication adherence, and self‐management of their disease condition. The interventions also provided educational material to the study participants that was used to help the patients get additional information related to their treatment and disease condition. Individual patients had an opportunity to discuss with their pharmacist at three time points in the 6‐month study period. Based on our findings, more contacts of patients with pharmacists have more effect on patients' awareness' of medication adherence. This finding was concordant with previous studies with interventions delivered on daily pharmacist contact, monthly pharmacist contact or telephone follow‐up have improved adherence to medication with different adherence rates (46–50).

Forgetfulness was the main reason that contributed to poor medication adherence in the pre‐intervention. This finding was consistent with studies done in the USA.^18^, ^26^ If HF patients forget to take their medications, they try to intentionally miss their medication or change the instructions provided by pharmacists.

In the post‐intervention phase, the cost of medication too expensive was the main reason that contributed to poor adherence. This finding was in line with a study conducted in Tanzania.^14^ Additionally, a study done in the USA^41^ reported a similar finding to our study which demonstrated that barriers to medication adherence among HF patients included limited communication with healthcare providers, forgetting to take daily medication, characteristics of medication (difficult schedule, frequent dosing, side effects, and difficulty swallowing), and cost of medication.

Patients who had co‐morbidity were 0.69 times less likely to be adherent to medications compared to patients who did not have co‐morbid disease during the pre‐intervention. This finding was comparable with studies done in Iran and the USA showing that the presence of co‐morbidity affects the medication‐taking behavior of HF patients leading to decreased medication adherence.^19^, ^23^, ^24^

In the post‐intervention, patients who took ≥5 drugs were 0.32 times less likely to be medication adherent compared to patients who had taken one drug. This finding is concordant with studies conducted in the USA and Iran, reaffirming that pill burden could decrease medication adherence.^19^, ^23^

There are some limitations in the current study. The study is single‐centered with a short duration of intervention. Medication adherence was measured by a self‐reported measure of adherence, which could have caused an overestimation of adherence. Despite the above limitations, the study shows tangible evidence of the effect of pharmacist intervention on medication adherence in Ethiopia with limited resources.

## 5 CONCLUSION

Interventions by pharmacists to educate HF patients about the nature of their disease and providing brochures to increase awareness of their medications have been shown to improve medication adherence.

## AUTHOR CONTRIBUTIONS

All authors of this study made a significant contribution to the conception, study design, execution, acquisition of data, analysis, and interpretation of the reported work, or in all these areas; took part in drafting, revising, or critically reviewing the article; gave final approval of the version to be published and agree to be accountable for all aspects of the work.

## FUNDING INFORMATION

There is no funder.

## CONFLICT OF INTEREST STATEMENT

The authors declare that they have no competing interests in this section.

## ETHICS STATEMENT

Ethical clearance of the study was obtained from Debre Berhan University Asrat Weldeyes Health Sciences Campus Ethical Review Board. All participants provided written consent to participate in the study through acceptance of the invitation. Each participant was informed about the objective of the study, and procedures of selection, and their rights to refuse were maintained.

## Data Availability

The datasets used and/or analyzed during the current study are available from the corresponding author upon reasonable request.
